# The Chemical Origin of Behavior is Rooted in Abiogenesis

**DOI:** 10.3390/life2040313

**Published:** 2012-11-07

**Authors:** Brian C. Larson, R. Paul Jensen, Niles Lehman

**Affiliations:** Department of Chemistry, Portland State University, P.O. Box 751, Portland, OR 97207, USA; E-Mails: bclarson@pdx.edu (B.C.L.); jensen3@pdx.edu (R.P.J.)

**Keywords:** behavior, chemistry, free will, RNA, origins of life

## Abstract

We describe the initial realization of behavior in the biosphere, which we term behavioral chemistry. If molecules are complex enough to attain a stochastic element to their structural conformation in such as a way as to radically affect their function in a biological (evolvable) setting, then they have the capacity to behave. This circumstance is described here as behavioral chemistry, unique in its definition from the colloquial chemical behavior. This transition between chemical behavior and behavioral chemistry need be explicit when discussing the root cause of behavior, which itself lies squarely at the origins of life and is the foundation of choice. RNA polymers of sufficient length meet the criteria for behavioral chemistry and therefore are capable of making a choice.

## 1. Introduction

Behavior is an integral feature of life. Behavior is manifest when a choice is possible, and a living entity responds to its environment in one of multiple possible ways. For relatively simple unicellular life, we tend to think of behavior as a deterministic response. Bacterial cells respond to a metabolite gradient in predictable ways, dictated by the biophysical processes of substrate uptake, second messenger activation, and flagella operation, for example. For relatively complex life such as sentient and conscious humans, we tend to think of behavior as a less predictable function—one that is enmeshed in the concept of free will [1].

Free will has been described as a modern *vitalism * [2], while Christian de Duve mentions, “we still know too little about the human mind to affirm categorically that it is a mere emanation of neural activity lacking the power to affect this activity.” [3] At this point, we are not going to indulge in the ageless philosophical debates of dualist and monist. Rather we consider behavior, defined as above and conceptualized in free will, the outcome of the interplay between genotype and phenotype based on evolution and stochasticity. If we consider the working definition of life as a self-sustaining chemical system able to evolve, then we can describe the manifestation and characteristics of this behavior as well as the progressive association with objects we call living opposed to a criterion to be alive.

As long ago as ~300 BCE the early atomist Epicurus hypothesized, in an attempt to refute the deterministic nature of the physical world and account for the behavior of choice, that all occurrences are due to small matter (atoms) colliding and interacting in voided space (kenos) and that the atoms are not restricted to straight lines; rather, they exhibit random swerves [4]. Epicurus introduced chance into the description of nature centuries before its being detailed by particle physics and quantum mechanics. Schrödinger expanded on the basis of choice saying, “The spatio-temporal process in the body of a living being which are in line with its intellectual activity and with its consciousness, or actions carried out in whatever way, are deterministic in a statistical sense, if not strictly deterministic in nature.” [5]

As conscious humans, our thoughts on choice and free will range between perceptions that a living entity functions as an absolute mechanism ultimately described by physical laws, and a constant personal awareness of making decisions of our own accord. Without anthropomorphizing molecules, we suggest that even the simplest of life must follow physical laws, but also have the ability to make a “choice” based on their environment and dependent on self-contained information.

Cashmore recently tackled this problem head on, and came to the conclusion that any atomic “swerve” was unable to lead to more than a perception of free will [2]. In his discussion of human behavior and free will he states, “in some ways, it might be more appropriate to replace ‘genetic and environmental history’ with ‘chemistry’—however, in this instance these terms are likely to be similar and the former is the one commonly used in such discussions.” Our premise is that this transition between genetic and environmental history and chemistry needs to be explicit when discussing the root cause of behavior, which itself lies squarely at the origins of life. During abiogenesis on the Earth, enough information became available that molecules accumulated the required chemical repertoire to make “choices” based on the environment, *i.e*., substrate uptake or folding pathways, and transitioned from a system in which chemicals had behavior to one in which chemicals could behave.

## 2. Chemical Behavior vs. Behavioral Chemistry

In order to address the ultimate source of “choice” we must differentiate between chemical behavior and behavioral chemistry. Chemical behavior is the essentially deterministic outcome of a chemical reaction among one or a few relatively small molecules. This is a term commonly used by chemists to describe the predicted products of a reaction under study, with the implicit assumption that any deviation from this outcome is simply a failing on our part to understand all the energetic facets of the molecules involved. In other words, chemical behavior is a deterministic process, one that becomes sharper the more we know about a system. A good example is the Haber production of ammonia: N_2_ + 3H_2_ ∧2NH_3_. Because of its historical and industrial importance, this is a very well studied reaction that has a deterministic outcome under typical reaction conditions. For example, at elevated temperatures and pressures, the negative enthalpy of this reaction (Δ*H* = −92.2 kJ/mol) will ensure that about 15% of nitrogen will be converted to ammonia, while the balance of the reactants will remain uncombined.

However, we would like to introduce the subtly different concept of behavioral chemistry. When the molecules involved are complex enough to attain a stochastic element to their structural conformation in such a way as to radically affect their function in a biological (evolvable) setting, then they have the capacity to behave, and the analysis of such molecular-level behavior would be described as behavioral chemistry. Behavioral chemistry is derived from two processes, either or both of which can be in operation at any given point in time. 

The first is the expression of quantum mechanical stochasticity “writ medium-sized” in the conformational energetics of macromolecules such as RNA and proteins. Here we mean that atomic swerves do not influence collections of small molecules in any meaningful way, but with the advent of polymeric macromolecules characteristic of life, the stochasticity begins to reassert an influence.

**Figure 1:**
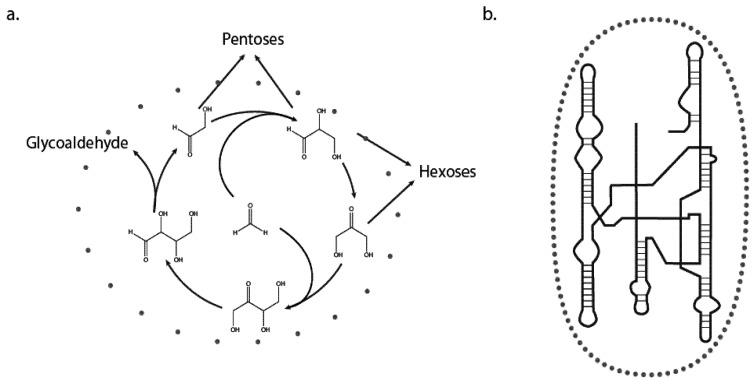
**(a)** The formose reaction. Incubation of formaldehyde and glycoaldehyde leads to the autocatalytic synthesis of hexoses and pentoses **(b)** Stick and line drawing representing RNA polymer secondary structure. In both panels grey circles represent the boundary of “self.” The boundary is ill defined for the formose reaction (a) but definite for the RNA (b).

At first glance one might claim that the distinction here is arbitrary, because even small molecules can adopt conformational variations in time that can affect their subsequent reactivities; think of the sugar pucker of glucose for example. However, this property does not maintain sufficient complexity to warrant behavior, and does not impact evolution. That said, complexity is not unique to living systems and can be seen in many non-living physical systems as well as in simple mathematical models. Complexity cannot solely determine behavior. A pertinent example of this is the formose reaction, whereby the incubation of the two simple precursors formaldehyde and glycoaldehyde can lead to the spontaneous synthesis of many sugars, including ribose. The formose reaction is both notoriously complex and has the feature of autocatalysis, making it of prebiotic importance [6]. It is in fact the best known example of a self-organized chemical cycle that bears on the origins of life but is absent of genetic material. Although the formose reaction can generate a wide range of reaction products that vary with the reaction conditions and phosphorylation states of the reactants, it fails to have the capacity to behave in the sense that we are defining. This is because the stochastic nature of the system, which originates in the quantum-mechanical nature of its component atoms, is mainly expressed in the set of side reactions that serve to convolute and disrupt the cycle, rather than to promote its continuation. The formose reaction, though autocatalytic, is essentially unbounded, meaning that it lacks a means to ensure survival of its “self.” The self is too poorly delineated (Figure 1a). 

Once Nature had the capacity to synthesize information-bearing macromolecules though, the stochasticity of the system became embodied into the “behavior” of the molecules because now there was the possibility that a molecule *was* the system! In essence, a system requires both a genotype and a phenotype to be able to display behavior. The “self” is now clearly defined (Figure 1b); however, it can be a single self-replicating molecule or a network of related cooperators. Here we are using the example of RNA as the informational polymer, but the same conclusions were to apply if other polymers, or even inorganic lattices or compositional sets of macromolecules such as lipids, were the ancestral genotypes. Clearly, the advent of compartmentalization (protocellular life) would further enhance the establishment of a bounded genotype [7], thereby firmly entrenching behavior. The key to this first aspect of behavioral chemistry is that the information is physically bondable such that the possible states of the system can be quantized (discrete). Once available, a boundary such as a membrane prevents informational loss, allowing for heredity and evolutionary change. This characteristic of life is not possible in an unbounded system such as the formose reaction. 

**Figure 2:**
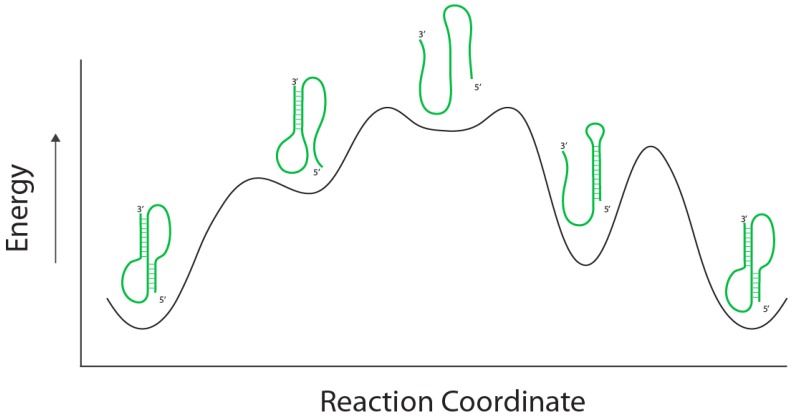
RNA Folding pathways. A RNA (green) can achieve a lowest energy fold through two separate folded intermediates. In the first (left-hand) intermediate, a less stable helix is formed 3’ end of the molecule followed by the formation of a helix in the 5’ portion to complete the psuedoknot. In the second (right-hand) intermediate a stem-loop is formed in the 5’ end of the molecule followed by hydrogen bond breakage to allow a helix to form in the 3’ portion of the psuedoknot. The energy states of the folds are dependent on the reaction coordinate of the folding pathway (black line).

The second process that allows behavioral chemistry is the real possibility that macromolecules end up in kinetic traps during their folding [8]. When the lifetimes of these traps are of the same order of magnitude as the time in which these molecules need to function, then a second type of behavioral expression becomes possible. An example of behavioral chemistry in operation is the metal-ion-dependent kinetic traps observed during the folding of many self-splicing RNA introns, such as the group I intron from the rRNA gene of *Tetrahymena* [9,10]. Another example is the equilibrium between pseudoknotted and non-psuedoknotted structures observed in the yeast telomerase RNA [11]. In this case, depending on the folding pathway, the reaction coordinate of the RNA folding trajectory can pass through one of two possible routes to the lowest energy fold; each pathway has a intermediate fold of distinct energy representing kinetic traps (Figure 2). Which pathway is taken depends on where the RNA happens to be prior to initiating folding, a state that depends in turn on the composite atomic thermal fluctuations of the composite atoms in the RNA. Empirical studies with single catalytic RNA molecules have revealed that the subtleties of their conformational dynamics are so complex that partially unfolded molecules possess a type of “memory” in that they tend to return to the folded state that they occupied prior to denaturation [12,13]. The important point here is that the molecule can be physically observed to be in one of the intermediate states, and thus an immensely large number of microscopic atomic ensemble states can be momentarily binned into two discrete macroscopic structures. The translation of quantum mechanical variation into alternative phenotypic states is the first manifestation of behavior at the chemical level.

## 3. From Behavioral Chemistry to Biological Behavior

**Figure 3:**
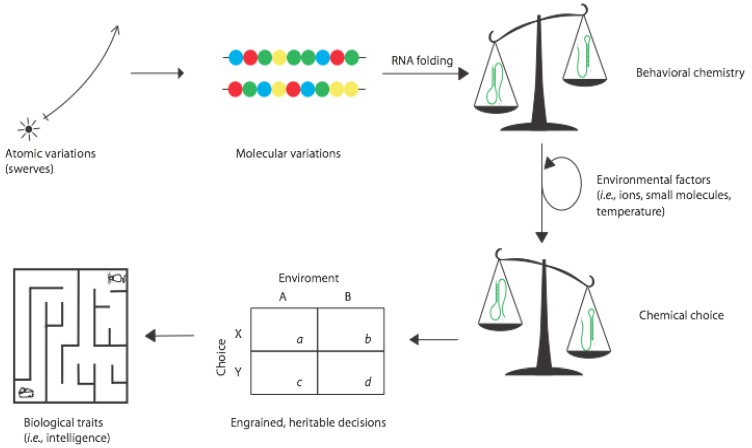
The evolution of behavior. Quantum mechanical and thermal fluctuations in single molecules get incorporated into informational polymers that can then display behavioral chemistry. Natural selection operating on these molecules leads to chemical choice, then to heritable decision making, and ultimately to behaviors ascribed to intelligent organisms.

At some point during the origins of life on the Earth, molecular systems acquired one or both of the conditions needed to satisfy the variations of chemical behavior described above. For the best understood (but still debated) case of RNA during a hypothetical RNA World, it is entirely possible that these variations typically overlapped ... that RNAs arose that had bounded information and were subject to kinetic folding traps. Such molecules could in principle respond to environmental conditions, interpret them [14], and adopt different conformations that represent crude choices. The steps to this stage, and from it to what we would recognize more as true organismal behavior, are diagrammed in Figure 3.

Once behavioral chemistry is possible in a system, the choices that molecules take would be subject to natural selection. We typically imagine the phenotypic state as being the target of selection, but imperfect heritability of phenotypes means that variable conformations can persist over generations, and some of these conformations can have negative fitness consequences. This can be observed empirically in populations of RNA evolving in the test tube [15]. However it is important to realize that the characteristic of being able to adopt—and chose among—alternative phenotypes is also subject to natural selection. Genotypes that confer only single phenotypes may be advantageous today, but during biogenesis the ability to choose in direct response to environmental perturbations could actually have conveyed a selective advantage. This would have set up a feedback loop between genotype and behavior as depicted in Figure 3.

Ultimately the ability of organisms––now collections of molecules with multiple genes and phenotypic products––to engrain reproducible behaviors would have been crafted by natural selection. The evolution of cellularity grants a more permanent boundary for the self, as discussed above. This second layer of bounding (beyond molecular covalence) adds richness to the spectrum of behavior by controlling which elements of the environment the self interacts with. Even single-celled organisms such as bacteria and protists could then express complex behavior, in the form of chemotaxis or phototaxis. For example, years of study on *E. coli* have revealed that this bacterial species expresses chemotaxis primarily through the actions of 15 proteins, such as specific chemoreceptors for amino acids. The functions of these proteins serve to bias an otherwise random walk powered by flagellar motion into a directional track toward certain ligands [16]. Directional and stabilizing selection operating on, say, ancestral alleles of these proteins could favor *E. coli* genotypes that migrated up a, say, serine concentration gradient when cellular serine stores were low. If this migration were not 100% coincident with a concomitant need to migrate up a, say, galactose concentration gradient, then a molecular choice becomes apparent. The advent of behavior, which is both flexible by definition and robust through genetic underpinnings was key in the establishment of a new phenomenon, evolvability [17], which in turn ensured the evolutionary persistence of behavior itself.

Eventually this led to the advent of behavioral ecology, with payoff matrices that could be tied to various behaviors [18]. While molecules could be capable of displaying behavioral chemistry that would be quantified in terms of a payoff matrix, empirical demonstrations of this are lacking, and it is has been more common to ascribe this level of complexity to cellular life and multicellular life in particular. 

Finally, however, organisms evolved intricate and convoluted means of interacting with their environments and displaying classical behavior, as in a rat choosing paths within a maze (Figure 3). Having developed the ability to speculate about what the future may bring and how best to survive potential threats, natural selection may have presented multicellular life with the ability to transcend strict determinism and make decisions of their own free will [1]. In terms of the behavior of an intelligent life form manifested in the act of making a choice, there are a few requirements to fulfill before this higher level meaning of choice is relevant in an evolutionary context:

(1) There must be at least two options available to the chooser. From a molecular perspective, this would be described in terms of what is allowed energetically (e.g., Figure 2). Perhaps importantly, we can think of options in terms of different goals, or in terms of different routes to reach one goal; both fit nicely into the context of what is energetically allowed.
(2) There must be a determination of one option as being optimal for some reason. For intelligent life forms, this generally comes after a cost/benefit analysis of some kind (Figure 3), which relies upon some previous exposure to or knowledge of the system around which a choice is being made. In the absence of any knowledge or exposure, a decision relies on some kind of unrelated bias, or is arbitrarily based upon probability (as in the case of a coin toss). For a molecular system lacking intelligence, the only real option is to fall to bias or probability, both of which are essentially yielding to energetic factors again.
(3) Some action must be taken towards realizing the choice being made. From a physical perspective, the objective determination of a choice being made is reliant upon some observable difference relative to the pre-choice state. In some sense, this is an extension to the organismal level of the molecular memory discussed above for RNA. Unless it can be communicated between intelligent beings in some kind of language, the only evidence of a choice is some subsequent action, which can be observed. Specifically, the choice or the ability to make it must have a fitness consequence.

Even at its highest level, behavior reverberates with the basic underlying properties of behavioral chemistry: one genotype can adopt multiple phenotypes, genotypes must sense and respond to their environments, and both the phenotype(s) and the ability to chose among them are subject to natural selection.

## 4. Free Will

The question remains whether the type of complex behavior we see in intelligent life is still proximally determined by an underlying behavioral chemistry, or whether the latter has been lost, having been subsumed into the biological operations of the cell long ago in evolutionary history. We essentially lack any understanding of how (mechanistically) the conscious mind interacts with the physical world. The fact that they do interact is most notably apparent in the context of our perceptions of the universe; we experience qualia, or feelings arising from sensations, which cannot be explained by the physical stimulus being sensed. For example, electronic transitions cause the emission of a photon of light, which causes a conformational change in an organic molecule in the eye, which causes an electrical signal in the brain—but we see “blue.” The physical steps leading to the concept of blue would exist without a conscious mind to perceive it, but the representation of photons of specific energies as a color would not. Examples of qualia such as seeing color, smelling smells, and feeling pain are instances where the physical world is causing an effect felt by the conscious mind via sensory organs. 

To organisms with the highest level of choice control, perceptions of other aspects of the physical world are not felt quite so directly, such as the feeling of passing through time. Humans understand time in reference to the frequency of a regularly repeating pattern, such as the rotation of the Earth, or Earth’s orbit around the Sun. We feel a sense of directionality in our existence, but we have to use objective visual cues to quantify it. From a physical standpoint, objective metrics that the universe is moving in a constant direction are scarce, (nuclear decay, entropy increasing, the universe expanding, *etc*.) and they are seemingly imperceptible *via* human’s five senses. We feel that we are moving through time, but we cannot explain or quantify that movement without relying on indirect cues such as watching a second-hand tick or the Sun travel through the sky. Some physical forces are still giving rise to the perceived directionality, but the information collected by our sensory organs serves only as a reference to quantify and conceptualize what we feel.

The interactions discussed above are all causal in that the physics of the environment give rise to the feelings being felt, clearly establishing that the physical world and the conscious mind interact, and that one can affect the other. Yet, scientists are keen to reject the concept of free will for lack of an objective parameter that can be studied empirically [2]. Indeed, the only evidence of free will that humans have is that we all feel as though we have it. It has been argued that this feeling arises from near constant observation that effects follow the “causes” which stem from our decisions – the concept of free will comes from recognizing a pattern. But the recognition that effect follows cause does not account for the fact that we perceive we have a measure of control over the cause. No such feeling of ownership arises from the recognition of the pattern that 1 + 1 = 2. It could be argued that the feeling of control over our decisions, which we all have, is a form of qualia which arises from *perceiving* the universe being actively changed by the choices we are making. Dennett argues that determinism does not imply inevitability, and that furthermore even if the physical reality were deterministic then there indeed are real options to life, not just apparent ones. His premise is that the process of natural selection has operated on an extant physics to create organisms with free will and with responsibilities for their own actions [1]. Here we extend this idea to the origins of life and attempt to place the origins of behavior into a chemical context. Further experimental work is needed to test some of the ideas put forth in this paper. 

## 5. Conclusions

Physical variations in molecules of a certain level of complexity can be trapped into quantum levels, and at this point, a transition from chemical behavior to behavioral chemistry can happen. This is the origin of all forms of behavior, and this transition was more-or-less coincident with the origins of life. The boundaries that define the self allow choice to transcend quantum mechanics and become engrained in chemistry and described better by statistical thermodynamics and Newtonian mechanics. Empirical studies that test the hypothesis that molecules can exhibit measurable properties that would satisfy the constraints of a payoff matrix will shed much light on this issue. Whether behavioral chemistry is still an operative force in the expression and evolution of organismal behavior is uncertain. Moreover, behavioral chemistry may have been an ultimate determinant of free will, but the notion of human perception clouds this issue.
